# Educating physicians on strong opioids by descriptive versus simulated-experience formats: a randomized controlled trial

**DOI:** 10.1186/s12909-022-03797-7

**Published:** 2022-10-26

**Authors:** Odette Wegwarth, Claudia Spies, Wolf-Dieter Ludwig, Norbert Donner-Banzhoff, Günther Jonitz, Ralph Hertwig

**Affiliations:** 1 Center for Adaptive Rationality, Max Planck Institute for Human Development, Lentzeallee 94, 14195 Berlin, Germany; 2 Heisenberg Chair for Medical Risk Literacy and Evidence-Based Decisions, Charité – Universitätsmedizin Berlin, Berlin, Germany; 3 Department of Anesthesiology and Operative Intensive Care, Charité – Universitätsmedizin Berlin, Berlin, Germany; 4 Drug Commission of the German Medical Association, Berlin, Germany; 5 Department of Primary Care, Phillips University Marburg, Marburg, Germany; 6 Berlin Chamber of Physicians, Berlin, Germany

**Keywords:** Strong opioids, Physicians’ risk perception, Physicians’ risk behavior, Drug safety, Risk literacy

## Abstract

**Background:**

Long-term prescriptions of strong opioids for chronic noncancer pain—which are not supported by scientific evidence—suggest miscalibrated risk perceptions among those who prescribe, dispense, and take opioids. Because risk perceptions and behaviors can differ depending on whether people learn about risks through description or experience, we investigated the effects of descriptive versus simulated-experience educative formats on physicians’ risk perceptions of strong opioids and their prescription behavior for managing chronic noncancer pain.

**Methods:**

Three hundred general practitioners and 300 pain specialists in Germany—enrolled separately in two independent exploratory randomized controlled online trials—were randomly assigned to either a descriptive format (fact box) or a simulated-experience format (interactive simulation).

**Primary endpoints:**

Objective risk perception (numerical estimates of opioids’ benefits and harms), actual prescriptions of seven therapy options for managing chronic pain.

**Secondary endpoint:**

Implementation of intended prescriptions of seven therapy options for managing chronic pain.

**Results:**

Both formats improved the proportion of correct numerical estimates of strong opioids’ benefits and harms immediately after intervention, with no notable differences between formats. Compared to description, simulated experience led to significantly lower reported actual prescription rates for strong and/or weak opioids, and was more effective at increasing prescription rates for non-drug-based therapies (e.g., means of opioid reduction) from baseline to follow-up for both general practitioners and pain specialists. Simulated experience also resulted in a higher implementation of intended behavior for some drug-based and non-drug-based therapies.

**Conclusions:**

The two formats, which recruit different cognitive processes, may serve different risk-communication goals: If the goal is to improve exact risk perception, descriptive and simulated-experience formats are likely to be equally suitable. If, however, the goal is to boost less risky prescription habits, simulated experience may be the better choice.

**Trial registration:**

DRKS00020358 (German Clinical Trials Register, first registration: 07/01/2020).

**Supplementary Information:**

The online version contains supplementary material available at 10.1186/s12909-022-03797-7.

## Background

There is insufficient evidence that strong opioids—defined as step III opioids on the World Health Organization analgesic ladder—are effective in the long term or superior to other analgesics in patients with chronic noncancer pain [1–4]. The absence of adequate scientific evidence is in striking contrast to the widespread use of opioids, in, for instance, Europe [5–9] and the United States [10, 11]. In Germany alone, about 80% of patients receiving strong opioids long term (> 3 months) have chronic noncancer pain. This is in conflict with a national evidence- and consensus-based clinical practice guideline [12] that cautions against the long-term use of strong opioids for this patient group and recommends that strong opioids be prescribed only after a thorough assessment of the benefits and harms, and with continual close monitoring.

One of several likely reasons behind the non-evidence-based practice of prescribing strong opioids long-term is that health care professionals and patients alike often have difficulties understanding medical statistics [13–23], leading to overly optimistic views of the benefit–harm ratios of medical interventions [24–26]. Transparent statistical formats (e.g., absolute instead of relative risks; [27] and visualizations (e.g., fact boxes; [28, 29] have been developed to clearly and transparently communicate medical risk information [23, 30]—however, they are not universally effective [24]. Research in cognitive psychology may provide an explanation for the boundaries of transparent communication. It has been shown that people’s perception and response to risks can be shaped by two different modes of learning: personal experience (e.g., taking medication and experiencing its consequences firsthand) and descriptive information (e.g., medical evidence and statistics in journal articles). An individual may behave as if they overestimate, underestimate, or correctly estimate a risk, depending on whether they experienced it themselves and/or received a description of it [31]. For instance, a doctor whose patient experienced a rare but severe side effect may deem the risk of this side effect to be significantly higher than its objective probability [32, 33]. Conversely, a doctor who prescribes a risky drug for many patients without any of them experiencing a rare side effect (samples from experience are often too small to include a rare but possibly cumulative risk) tends to act as if they underestimated or underweighted the risk [34–36]. In the absence of personal experience, descriptive risk information can have an excessive psychological influence on people’s risk perception and behavior (e.g., overenthusiasm for a drug). However, if an individual has both personal experience and descriptive information, experience—which usually feels more authoritative, concrete, transparent, and trustworthy (regardless of whether it actually is; [37])—is likely to prevail over description [38].

Some recent evidence suggests that simulating experience can help combat undesired behavioral consequences of different states of knowledge about a risk [31]. For instance, investors’ risk perception and behavior improved when they learned about the volatility and risk of a stock market investment by experiencing a past return distribution in an interactive simulation, relative to when they were shown static graphs depicting the investment’s past returns [39]. Similarly, some studies suggest that laypeople are better at estimating the positive predictive value of a diagnostic test when they receive risk information in an experience-based format than when they receive it in a descriptive format [40]; however, other studies do not support this finding [41, 42]. Until now there has been no investigation into how these two modes of learning about risks impact risk perception and behavior in the area of drug safety. As a consequence, it is unclear whether simulated experience could be harnessed to help professionals and patients make sound decisions about the use of potentially risky drugs [43].

To find out about the effects of the two modes of learning about risks and the potential existence of a description–experience gap—the “systematic discrepancies between description- and experienced-based choices” (Wulff et al., 2018, p. 140)—in the domain of drug safety, we conducted four explorative randomized controlled trials (RCTs) under the umbrella of the ERONA project. The RCTs investigated four groups involved in the long-term administration of strong opioids: general practitioners (GPs), physicians specialized in pain therapy (pain specialists), patients with chronic (≥ 3 months) noncancer pain, and pharmacists who regularly dispense narcotic substances. Here, we report results from the ERONA trials with physicians—GPs and pain specialists—on the effects of educative interventions featuring either a simulated-experience format (interactive simulation) or a descriptive format (fact box) on their objective risk perception, their intended prescription behavior, and their actual prescription behavior 9 months later.

## Methods

The ERONA project was funded by a grant from the German Federal Ministry for Health under the guideline “Risk perception and risk behavior among stakeholders involved in settings of drug safety concern.” The designs and methods have been described in detail and published in a peer-review journal as a study protocol [44] that has not since been amended. The trial was registered at the German Clinical Trials Register (DRKS00020358) and trial information was made public on the Open Science Framework (OSF). The trial adheres to the CONSORT checklist. The study is based on an exploratory independent RCT with two parallel online intervention arms. Randomization to either intervention was achieved by simple randomization: Participants were assigned to either group by pure chance (like flipping a coin). Participants did not know which intervention they received. Data were collected before intervention at baseline (T0, regarded as control condition), immediately after intervention (T1), and 9 months after intervention (T2). The Institutional Ethics Board of the Max Planck Institute for Human Development, Berlin, Germany approved the study (Ethic Approval ID: A 2020–05).

### Intervention

**Fig. 1:**
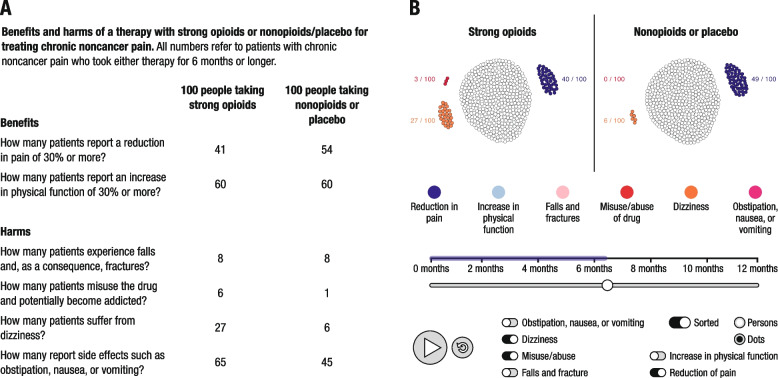
Intervention formats: **A** descriptive format; **B** simulated-experience format

A fact-box format was used as the descriptive intervention and an interactive simulation as the simulated-experience intervention (Fig. 1). Both risk-education interventions presented information on the benefit–harm ratio associated with the long-term administration of strong opioids in patients with chronic noncancer pain. Benefits and harms were presented in frequencies, adjusted to the same denominator (here: per 100 people), and compared with a control group (here: nonopioids or placebo). Numerical estimates of the magnitude of benefits and harms were based on a systematic rapid review conducted for this RCT by the Institute for Evidence in Medicine (for the Cochrane Germany Foundation; [3]).

Fact boxes (Fig. 1A) typically present information on the benefits and harms of each treatment in a tabular, static form. In order to make the descriptive intervention more comparable to the simulated-experience intervention in terms of interactivity, we modified the fact box using the Mouselab methodology [45] (www.mouselabweb.org). That meant that numerical information about each benefit and harm was concealed and participants had to move the mouse pointer over the cells of the fact box in order to access the information.

The interactive simulation presented information on the benefits and harms of strong opioids and of nonopioids/placebo interactively and sequentially (Fig. 1B). Participants could press the play button at the bottom of the simulation to directly observe how the benefit–harm ratio would change over 12 months of taking strong opioids. Moving the horizontal time frame slider allowed participants to view particular moments in time. The simulation also featured interactive filter functions that allowed participants to explore each outcome separately by activating or deactivating the respective outcome buttons.

In both interventions, participants were not limited in the amount of time they could engage with the educative interventions.

### Survey questionnaire and outcome measures

The interventions were implemented as an online survey. Before starting the survey, participants' eligibility was first determined by a set of screener questions to ensure that physicians belonged to the target disciplines and that they regularly prescribe strong opioids to chronic noncancer pain patients; they also provided demographic information (age, gender, years in practice, region of practice). Participants who passed the screener questions were told that the study aimed to better understand physicians’ evaluation of strong opioids’ benefits and harms and their prescription habits. They were made explicitly aware of socially desirable response behavior and were asked to answer as truthfully as possible.

The two primary endpoints surveyed were objective risk perception, measured at baseline (T0) and immediately after intervention (T1), and reported actual prescription behavior, measured at T0 and T2, for pain medications (strong opioids, weak opioids—defined as step II opioids on the World Health Organization analgesic ladder, and analgesics) and therapy alternatives to strong opioids (multimodal therapy, physiotherapy, psychotherapy, opioid reduction). Objective risk perception was measured using six questions, each requiring participants to provide a numerical estimate for one of six outcomes (benefits/harms) presented in the intervention: significant reduction (≥ 30%) of pain; significant increase (≥ 30%) of physical function; risk of falls and fractures; risk of opioid misuses; risk of dizziness; and risk of obstipation, nausea, and vomiting (Fig. 1). For example, for the benefit “reduction in pain” we asked: “How many people out of 100 taking strong opioids for 6 months or longer do you think will experience a reduction in pain of at least 30%?” Apart from the specific benefit or harm addressed, the wording of the questions was the same throughout. Physicians’ prescription behavior at baseline (T0) was investigated by asking them to imagine the patients with chronic noncancer pain they had treated within the past 12 months and to indicate the proportion of seven pain therapy options (strong opioids, weak opioids, nonsteroid anti-inflammatory drugs [NSAID], multimodal therapy, physiotherapy, psychotherapy, and opioid reduction measures) they had prescribed per 100 patients by moving a slider between 0 and 100 percent. To investigate the influence of interventions on physicians’ actual prescription behavior after 9 months (T2), we presented them with their responses on their intended prescription behavior—measured at T1—and asked them to indicate any increase or decrease in their actual prescriptions relative to their intended prescription behavior by moving the slider accordingly.

A secondary endpoint was physicians’ intended prescription behavior, measured immediately after intervention (T1). In order to probe intended prescription behavior, each physician was presented with their own responses on therapies from T0 and asked to indicate any intended increase or decrease in their prescription behavior for each of the seven alternatives by moving a slider accordingly. We also assessed participants’ medical risk literacy as a moderator variable by administering an adapted version of the validated Critical Risk Interpretation Test (CRIT; [46]). The phrasing of the survey was piloted with 11 German GPs and pain specialists who prescribe strong opioids to ensure readability and relevance, and revised using their feedback.

### Participants

The market research institution IPSOS Health (Nuremberg, Germany) sourced the two samples of populations of GPs and pain specialists by using publicly available lists of medical professionals of the National Association of Statutory Health Insurance Physicians (KBV) and public directories. The two populations were chosen because they represent two key disciplines that provide opioids to patients with chronic noncancer pain in ambulant care. Although we report findings for both disciplines in this manuscript, we do not compare the results because the two disciplines deal with different patient groups with different degrees of severity of pain in daily care (which was also the rationale for investigating them as separate entities in the ERONA project).

The trial per specialty required 300 participants (150 per intervention arm) in order to detect a 15% difference between intervention conditions (two-sided alpha of 5%, power of 80%; for details, see [44]). The rationale for using 15% as a difference was based on effects found in other studies [40, 47, 48] comparing current standard risk information with one or the other of the evidence-based risk formats (fact box, simulation) used in our trial.

IPSOS started enrolment for the first wave (T1) in April 2020 and concluded the first wave in August 2020. Enrolment for the 9-month follow-up (T2) began in January 2021 with the participants who started first in the first wave and was completed in April 2021.

IPSOS approached 3,431 GPs and 5,389 pain specialists in order to obtain 300 complete sets of data per specialty for T0 and T1. Nine months after the first wave, participants were approached again to obtain data for T2. For T2, 214 of the 300 GPs (fact box: *n* = 110, simulation: *n* = 104) and 212 of the 300 pain specialists (fact box: *n* = 99, simulation: *n* = 113) participated in the 9-month follow-up (CONSORT flow charts, Supplemental Materials). Written informed consent was obtained from all participants prior to the study. Participants were remunerated with 40 euros.

### Analyses

For the primary analysis, we compared objective risk perception and prescription behavior between intervention groups. To analyze the effect of interventions on the primary endpoint “objective risk perception,” we compared the proportion of correct estimates per benefit and harm at T1, the absolute change from baseline in the proportion of correct estimates at T1, and the total number (minimum: 0, maximum: 6) of correct estimates across all benefit and harm endpoints at T1 between the two intervention groups. We counted participants’ risk estimates (e.g., for reduction of pain: 41 people out of 100) as correct if the numerical value fell within a ± 15% relative margin of error around the respective point estimate (e.g., for pain, between 34 and 46). Differences were assessed using a χ^2^ test. To evaluate the influence of the interventions on the second primary endpoint “prescription behavior,” we investigated for mean differences in the reported proportions of actual prescriptions for each of the seven therapy options at follow-up (T2) and for differences in the mean change from baseline (T0) for each of the prescriptions at T2 between the two intervention groups using ANCOVA models that included the baseline as a covariate. The differences between groups in prescription rates are therefore presented as a baseline adjusted mean difference estimates with 95% confidence intervals.

For the secondary analysis, we studied the implementation of physicians’ intended behavior [49]. We measured implementation of intended behavior by determining whether the actual prescription behavior at T2 equaled or positively exceeded the intended behavior reported at T1. For “prescription of WHO-III-opioids” and “prescription of WHO-II-opioids,” prescription rates at T2 positively exceeded intended behavior if they were lower than intended at T1; for the remaining five therapy options, prescription rates positively exceeded intended behavior if they were higher than intended. If implementation positively exceeded or was equal to the intended behavior, the value was labeled “1”; otherwise, “0.” Differences between groups were then assessed using a χ^2^ test.

We also investigated the influence of medical risk literacy and demographics on participants’ change in objective risk perception (calculated as change in sum of correct estimates across all six benefits and harms) and change in prescription behavior (calculated as sum of changes in prescription proportion between T0 and T2 for each therapy alternatives). To determine participants’ medical risk literacy, we calculated a sum score of correct responses across the five questions asked by the adapted version of the CRIT test; the higher the sum, the higher the assumed medical risk literacy. Independent predictors (e.g., medical risk literacy) of physicians’ likelihood to change their risk perception and prescription behaviors were then analyzed using regression analysis.

Due to dropout rates of 28.7 percent (GPs) and 29.3 percent (pain specialists) between T1 and T2, we performed dropout analysis using a **χ**^2^ test (categorical variables) and an independent t-test (continuous variables) and compared baseline differences for gender, years of profession, region of practice, the absolute number of correct risk estimates, and reported prescriptions for each of the seven therapy options at baseline. In addition, we investigated for differences in the increase of correct risk estimates from T0 to T1 and the intended prescription for each of the seven therapy options at T1. Where we recognized baseline differences in prescription rates between non-dropouts and dropouts, we used regression imputation to estimate corrected mean follow-up prescription rates. For the primary questions of group differences in T2 prescription rates and in prescription rate changes, the dropout cases carry no information, which is why we used complete-case analysis. The differences were estimated as baseline adjusted mean difference estimates via linear ANCOVA models. For the means and SDs of T2 prescription rates and T2 prescription rate changes, however, dropouts may indeed contain information: As the baseline differences translate into follow-up bias through an association of baseline values with follow-up values (which is plausible and also shows in the linear models), unbiased estimates for the follow-up means are computed using regression imputed follow-up values from the models for all options for which we detected differences between dropouts and non-dropouts (i.e., for pain specialists: “NSAID prescription” and “means of opioid reduction”).

Data were stored and analyzed with IBM SPSS Statistics 26. To control for nonresponse bias [50], we compared the demographic characteristics of survey respondents and those participants who left the survey prematurely [51, 52]. The online questionnaire did not permit item nonresponse; data sets were therefore complete.

## Results

For GPs and pain specialists, intervention groups did not differ in terms of distribution of age, gender, years in practice, and region of practice. Table 1 reports the distribution of age, gender, years in practice, and region of practice for all GPs and pain specialists who finished the survey (respondents) and for those who abandoned the survey prematurely (nonrespondents). Using a ± 5% margin of difference, respondents tended to be older and more experienced in terms of years in practice than nonrespondents. There were more male than female pain specialists, and more GPs were from the south of Germany than from other regions.

**Table 1:** Demographic characteristics of respondents and nonrespondents

	General practitioners (*n* = 300)		Pain specialists (*n* = 300)	
	Respondents	Non-respondents	Respondents	Non-respondents
	%^*^	%^*^	%^*^	%^*^
Female	38.0	40.0	35.7	40.3
Age (in years)				
20–39	11.0	10.9	6.0	6.9
40–59	63.0	69.1	74.7	79.2
60–79	26.0	20.0	19.3	13.9
Years in practice				
< 10	10.3	9.1	5.0	6.9
10–19	27.3	34.5	39.0	44.4
20–29	41.3	38.2	41.0	41.7
30–39	20.7	18.2	15.0	6.9
≥ 40	0.3	0.0	0.0	0.0
Region of practice in Germany				
North	21.3	30.9	23.0	20.8
East	27.3	23.6	26.0	26.4
South	23.7	14.5	20.7	19.4
West	27.7	30.9	30.3	33.3

^*^ Percentages are rounded and may not total 100

### Effect of interventions on objective risk perception

There were no meaningful differences between GPs who received the descriptive or the simulated-experience format in terms of proportion of correct estimates per outcome after intervention (T1; Table 2), the increase from baseline in the proportion of correct estimates at T1 (Table 2), or total amount of correct estimates (maximum: 6 correct estimates) across all outcomes (χ^2^ [6; *n* = 300] = 4.4, *p* = 0.619).

**Table 2:** Influence of descriptive versus simulated-experience formats on correct risk estimates of the benefits and harms of long-term administration of strong opioids for general practitioners and pain specialists

	Proportion of correct estimates (%) at T1			Absolute increase from baseline (T0) in the proportion (%) of correct estimates at T1		
	Fact box	Simulation	p* (Cramer’s V) 95% CI**	Fact box	Simulation	p* (Cramer’s V) 95% CI
**General practitioners**						
Reduction in pain	8.7	4.7	.166 (.080) 0.76 – 4.52	7.3	4.7	.331 (.056) 0.63 – 3.94
Increase in physical function	48.7	42.7	.297 (.060) 0.89 – 1.46	22.7	17.3	.330 (.086) 0.83 – 2.07
Risk of falls/ fractures	19.3	19.3	1.00 (.000) 0.63 – 1.59	10.7	9.3	.700 (.022) 0.68 – 1.57
Risk of misuse/ addiction	44.7	47.3	.643 (.027) 0.74 – 1.21	22.7	22.0	.758 (.043) 0.58 – 2.26
Risk of dizziness	46.0	47.3	.817 (.013) 0.76 – 1.24	20.0	18.7	.803 (.038) 0.67 – 1.70
Risk of nausea, obstipation, or vomiting	30.7	29.3	.801 (.015) 0.74 – 1.48	15.3	14.0	.896 (.027) 0.63 – 1.89
**Pain specialists**						
Reduction in pain	9.3	6.7	.395 (.049) 0.64 – 1.27	9.3	6.0	.342 (.085) 0.69 – 3.48
Increase in physical function	40.7	42.7	.815 (.014) 0.73 – 1.27	24.0	17.3	.292 (.091) 0.88 – 2.17
Risk of falls/ fractures	30.0	14.7	.001*(.184) 1.29 – 3.23	14.7	8.7	.236 (.098) 0.88 – 3.23
Risk of misuse/ addiction	45.3	37.0	.090 (.151) 1.10 – 1.99	13.3	13.3	.954 (.018) 0.56 – 1.78
Risk of dizziness	62.0	62.0	1.00 (.000) 0.84 – 1.19	14.7	16.7	.318 (.087) 0.52 – 1.49
Risk of nausea, obstipation, or vomiting	48.7	40.7	.163 (.080) 0.93 – 1.54	15.3	15.3	.929 (.022) 0.59 – 1.70

T0 = investigation at baseline, T1 = investigation immediately after the interventions

^*^Significance level is two-tailed and based on a chi-square test

^**^ 95% confidence interval (CI) for risk ratio between intervention groups

Findings for pain specialists mostly mirrored those for GPs. There were no meaningful differences between the two intervention groups in terms of the increase from baseline in proportion of correct estimates per outcome at T1 (Table 2), or the total sum of correct estimates across all outcomes (χ^2^ [6; *n* = 300] = 9.5, *p* = 0.146) at T1. The proportion of correct estimates per outcome differed for one harm outcome between intervention groups: Pain specialists who saw the fact box had a higher proportion of correct estimates than did those who saw the simulated experience for “risk of falls and factures” at T1 (30.0% vs. 14.7%, χ^2^ [1; *n* = 300] = 10.2, *p* = 0.001, 95% CI: 1.29 – 3.23). No differences were found for the remaining five benefit and harm estimates between intervention groups (Table 2).

**Fig. 2:**
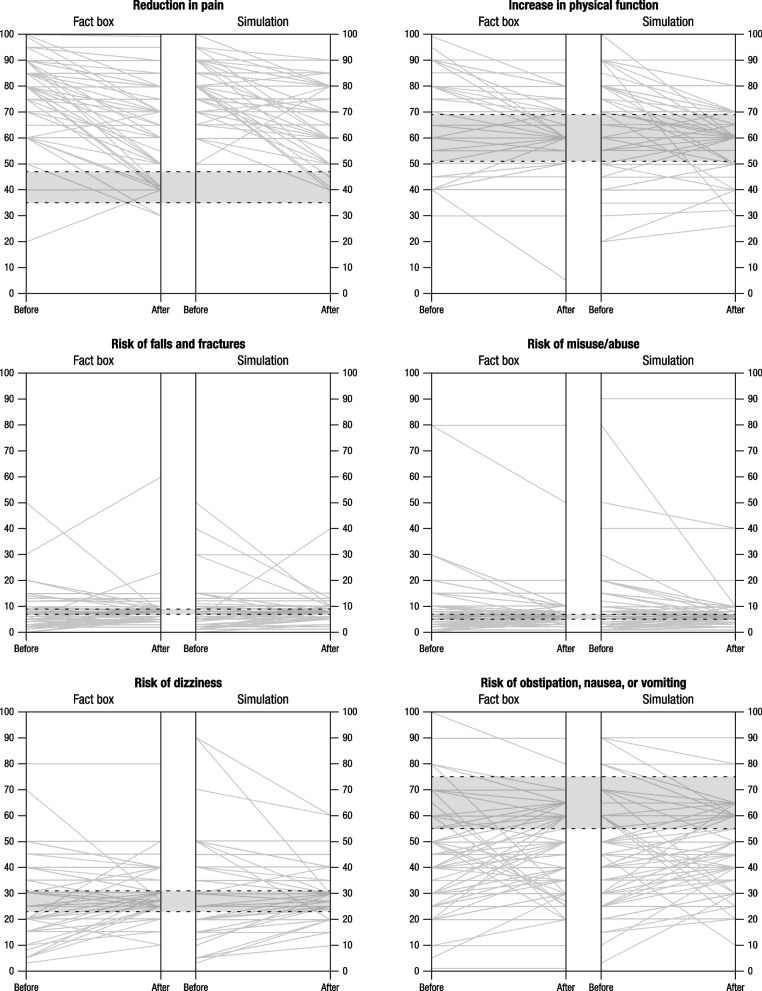
General practitioners’ risk estimates for benefits and harms at baseline (T0) and after intervention (T1). Note: The gray area within the dashed lines shows correct estimates falling within the ± 15% margin of error

**Fig. 3:**
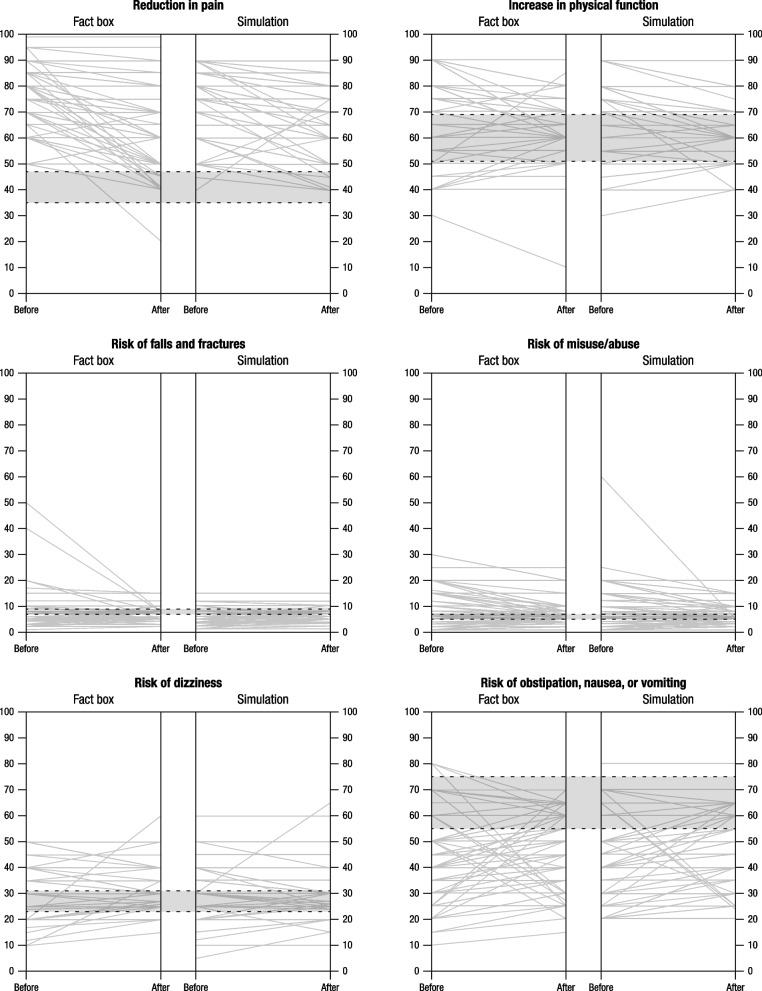
Pain specialists’ risk estimates for benefits and harms at baseline (T0) and after intervention (T1). Note: The gray area within the dashed lines shows correct estimates falling within the ± 15% margin of error

Figures 2 and 3 illustrate individual changes in risk estimates of GPs and pain specialists, respectively, before and after intervention and for each outcome by intervention; correct estimates (within a ± 15% margin of error) are shown in the gray area.

### Effect of interventions on GPs’ actual prescription behavior and their implementation of intended behavior

Intervention groups did not differ in their prescription behavior for any of the seven therapy options at baseline (Table 3). After intervention, however, GPs in the simulated-experience condition were significantly less likely than GPs in the descriptive condition to prescribe strong opioids (mean group difference [95%-CI] -1.89 [-3.22; -0.55] percentage points, *p* = 0.006) and weak opioids (mean group difference [95%-CI] -4.89 [-6.4; -3.39] percentage points, *p* < 0.001), and were more likely to prescribe less risky options: NSAID (mean group difference [95%-CI] 2.24 [0.09; 3.58] percentage points, *p* = 0.001), psychotherapy (mean group difference [95%-CI] 1.24 [0.38; 2.1] percentage points, *p* = 0.005), and means of opioid reduction (mean group difference [95%-CI] 2.3 [-0.97; -3.63] percentage points, *p* = 0.001) at T2 (Table 4).

**Table 3:** Differences in prescription behaviour at baseline T0 for general practitioners and pain specialists who also participated at T2/ significant level is based on an independent two-tailed t-test

**General Practitioners**				
***Prescription at baseline (T0)***	*Fact box* *(n* = *110)*	*Simulation* *(n* = *104)*	*p*	*Cohen‘s d*
WHO-III Opioids (strong)	23 ± 10.8	22.1 ± 10.9	.531	0.086
NSAID	84.8 ± 15.7	87.6 ± 14	.172	-0.188
WHO-II Opioids (weak)	12.3 ± 9.8	13.5 ± 11.5	.398	-0.116
Multi-Modal Therapy	18 ± 13.1	16.8 ± 13.8	.503	0.092
Physiotherapy/ Endurance Sports etc	67.2 ± 22.7	68 ± 21.7	.797	-0.035
Psychotherapy	29.9 ± 24.3	29.1 ± 23.4	.824	0.031
Means of Opioid Reduction	27.4 ± 22.4	26.1 ± 22.6	.675	0.057
**Pain Specialists**				
***Prescription at baseline (T0)***	*Fact box* *(n* = *99)*	*Simulation* *(n* = *113)*	*p*	*Cohen ‘s d*
WHO-III Opioids (strong)	26.7 ± 12.2	258 ± 11.6	.586	0.075
NSAID	74.9 ± 21.2	80 ± 20.6	.078	-0.244
WHO-II Opioids (weak)	16.2 ± 13.5	14.9 ± 11.8	.459	0.102
Multi-Modal Therapy	28.2 ± 17.3	26.2 ± 16.8	.395	0.117
Physiotherapy/ Endurance Sports etc	65.2 ± 19.6	64 ± 19.1	.676	0.058
Psychotherapy	50.4 ± 31.8	44.1 ± 30.9	.143	0.202
Means of Opioid Reduction	46.9 ± 31.7	39 ± 30	.064	0.256

**Table 4:** Between-group-differences in actual proportions of prescription per therapy at T2 and in changes from baseline in prescription proportions at T2 for general practitioners and pain specialists

	Mean (SD) proportion of prescription per 100 patients at T2		Change from baseline in Mean (SD) proportion of prescription per 100 patients at T2		
	Fact box	Simulation	Fact box	Simulation	p* Baseline adjusted mean difference [95% CI]
**General practitioners**	(*n* = 110)	(*n* = 104)	(*n* = 110)	(*n* = 104)	
Strong opioids	20.8 (9.4)	18.3 (9.2)	– 2.2 (5.1)	– 3.9 (6.3)	0.006* –1.89 [– 3.22; – 0.55]
Nonsteriod anti-inflammatory drugs (NSAID)	83.8 (15.4)	88.5 (11.6)	– 1.0 (3.5)	0.9 (6.8)	0.001* 2.24 [0.9; 3.58]
Weak opioids	13.7 (10.5)	9.7 (9.1)	1.4 (4.6)	– 3.8 (7.4)	< 0.001* – 4.89 [– 6.4; – 3.39]
Multimodal therapy	17.9 (13.0)	17.3 (13.8)	– 0.2 (3.6)	0.5 (2.5)	0.129 0.65 [–0.19;1.48]
Physiotherapy, endurance sports	68.2 (21.4)	68.2 (21.2)	1.0 (5.6)	0.2 (3.3)	0.239 – 0.71 [–1.91; 0.48]
Psychotherapy	29.5 (23.8)	30.0 (22.8)	– 0.4 (3.6)	0.9 (2.9)	0.005* 1.24 [- 0.38; 2.1]
Means of opioid reduction	27.0 (22.6)	28.0 (22.1)	– 0.4 (5.4)	1.9 (4.5)	0.001* 2.3 [– 0.97; 3.63]
**Pain specialists**	(*n* = 99)	(*n* = 113)	(*n* = 99)	(*n* = 113)	
Strong opioids	25.3 (11.5)	22.5 (11.1)	– 1.4 (3.8)	– 3.3 (5.4)	0.002* –1.98 [– 3.2; –0.762]
Nonsteriod anti-inflammatory drugs (NSAID)	76.5 (20.2) ^§^	80.8 (18.4) ^§^	– 1.2 (4.0) ^§^	– 0.3 (4.9) ^§^	0.080 1.01 [– 0.12: 2.13]
Weak opioids	16.8 (12.6)	12.6 (10.3)	0.6 (6.5)	– 2.3 (6.7)	< 0.001* – 3.19 [– 4.82; –1.56]
Multimodal therapy	27.7 (16.7)	26.2 (16.8)	– 0.5 (2.7)	0.1 (4.2)	0.393 0.41 [– 0.54;1.37]
Physiotherapy, endurance sports	66.0 (18.6)	65.2 (19.1)	1.1 (4.9)	1.1 (4.2)	0.715 0.23 [– 1;1.45]
Psychotherapy	50.6 (30.6)	44.3 (30.1)	0.2 (3.3)	0.2 (3.6)	0.584 – 0.25 [– 1.16; 0.66]
Means of opioid reduction	42.2 (30.2) ^§^	43.5 (29.8) ^§^	– 0.6 (7.3) ^§^	6.5 (14.2) ^§^	< 0.001* 6.41 [3.35; 9.48]

T2 = mean prescription behavior across all respondents of intervention group at 9-month follow-up; T0 = mean prescription behavior across all respondents of intervention group at baseline; mean and standard deviation (SD) of number of patients out of 100 with chronic noncancer pain being prescribed the respective treatment

^*^ Significance level is based on ANCOVA models with adjustment for baseline values

^§^ Mean T2 rates and changes for NSAID and means of opioid reduction in pain specialists were computed with imputed values due to significant differences between dropouts and non-dropouts, *n* = 150 + 150

Compared to description, simulated experience also resulted in a higher propensity of GPs to implement their intended behavior (T1) at the 9-month follow-up (T2; Supplementary Table S1) for four of seven therapy options: strong opioids (93.3 vs. 98.2; *p* = 0.013), weak opioids (79.1 vs. 94.2; *p* = 0.001), multimodal therapy (80.9 vs. 88.0; *p* = 0.041), and means of opioid reduction (82.8 vs. 92.2; *p* = 0.003). For the remaining three options we found no difference in the implementation of intended behavior between the two intervention groups (further details in Supplementary Table S1).

### Effect of interventions on pain specialists’ actual prescription behavior and their implementation of intended behavior

Again, intervention groups did not differ in their prescription behavior at baseline (Table 3). After intervention, pain specialists presented with the simulated experience were significantly less likely than pain specialists presented with the fact box to prescribe strong opioids (mean group difference [95%-CI] 1.98 [-3.2; -0.76] percentage points, *p* = 0.002) and weak opioids (mean group difference [95%-CI] -3.19 [-4.82; -1.56] percentage points, *p* < 0.001), and significantly more likely to prescribe means of opioid reduction (mean group difference [95%-CI] 6.41 [3.35; 9.48] percentage points, *p* < 0.001) at T2 (Table 4). That is, compared to the fact-box condition, the simulated-experience condition resulted in a significantly greater mean reduction from T0 to T2 in prescriptions for strong and weak opioids, and a significantly greater mean increase in the prescription of means of opioid reduction (Table 3).

Likewise, compared to description, simulated experience resulted in a higher propensity of pain specialists to implement their intended behavior (T1) at the 9-month follow-up (T2; Supplementary Table S1) for three of seven therapy options: strong opioids (91.9 vs. 98.2; *p* = 0.031), multimodal therapy (77.8 vs. 89.4; *p* = 0.022), and means of opioid reduction (86.9 vs. 95.6; *p* = 0.023). For the remaining four options there were no differences in the implementation of intended behavior between groups (Supplementary Table S1).

### Influence of medical risk literacy and demographics on change in objective risk estimates and prescription behavior

Changes in physicians’ objective risk estimates and prescription behavior were significantly influenced by medical risk literacy. The higher their medical risk literacy, the more likely physicians were to update their beliefs towards an accurate estimation of the benefits and harms after intervention (GPs: odds ratio [OR]: 1.46, 95%; CI: 1.06–1.99; *p* = 0.020; pain specialists: OR: 1.62, 95%; CI: 1.15–2.28; *p* = 0.006). We found a reverse effect of medical risk literacy on physicians’ reported change in prescription behavior: Higher medical risk literacy was associated with a lower likelihood of changing initially reported prescription rates (GPs: OR: 0.63, 95%; CI: 0.46–0.87; *p* = 0.006; pain specialists: OR: 0.60, 95%; CI: 0.42–0.87; *p* = 0.007). The reverse effect may be due to the fact that GPs and pain specialists with higher risk literacy (≥ 3 answers correct) at baseline reported significantly fewer prescriptions of strong opioids and NSAID, as well as significantly higher prescription rates for the three non-drug-based therapy options (multimodal therapy, psychotherapy, and means of opioid reduction) compared to those with lower medical risk literacy (≤ 2 answers correct). Physicians with higher medical literacy may therefore have simply had fewer opportunities to change prescription rates between T0 and T2.

Neither gender nor years in practice was associated with the likelihood of GPs or pain specialists to change risk estimates on strong opioids’ benefits and harms, nor were they associated with changes in prescription rates among GPs. However, two demographic aspects were linked with changes in prescription rates among pain specialists: Being male (OR: 1.91, 95%; CI: 1.02–3.57; *p* = 0.043) and having fewer years in practice (OR: 0.46, 95%; CI: 0.27–0.81; *p* = 0.008) increased a pain specialist’s likelihood of changing their initial prescription after intervention.

### Analysis of differences between participants completing or not completing follow-up survey

Among GPs, there were no differences between those who did or did not complete the follow-up survey in terms of gender (χ^2^ [1; *n* = 300] = 0.195, *p* = 0.659, Cramer’s V = 0.026, 95% CI: 0.89 – 1.20), years in profession (χ^2^ [4; *n* = 300] = 3.386, *p* = 0.459, Cramer’s V = 0.106), region of practice (χ^2^ [3; *n* = 300] = 2.060, *p* = 0.560, Cramer’s V = 0.083), number of correct risk estimates provided for each of the six benefits and harms at baseline (χ^2^ [4; *n* = 300] = 5.396, *p* = 0.249, Cramer’s V = 0.134), or reported proportional prescriptions for any of the seven therapy options (Supplementary Table S2) at baseline. Also, there were no differences in increase of the amount of correct risk estimates from T0 to T1 (χ^2^ [6; *n* = 300] = 7.186, *p* = 0.517, Cramer’s V = 0.155) or reported intended proportional prescriptions for any of the seven therapy options (Supplementary Table S2) after intervention at T1.

Among pain specialists, the results were similar: There were no differences between those who had completed the follow-up survey and those who had not in terms of gender (χ^2^ [1; *n* = 300] = 0.010, *p* = 0.918, Cramer’s V = 0.006, 95% CI: 0.67 – 1.42), years in profession (χ^2^ [3; *n* = 300] = 1.410, *p* = 0.703, Cramer’s V = 0.069), region of practice (χ^2^ [3; *n* = 300] = 4.770, *p* = 0.189, Cramer’s V = 0.126), number of correct risk estimates at baseline (χ^2^ [4; *n* = 300] = 8.085, *p* = 0.089, Cramer’s V = 0.164), increase in correct risk estimates from T0 to T1 (χ^2^ [6; *n* = 300] = 5.742, *p* = 0.570, Cramer’s V = 0.138), or initial (T0) and intended (T1) prescription behavior for five out of seven therapy options (Supplementary Table S2). There were differences in the means of prescription proportions at baseline (T0) and at T1 for NSAID, with higher prescription rates among those who dropped out compared to those who participated in the follow-up, and for opioid reduction, with lower prescription rates among those who dropped out (Supplementary Table S2).

## Discussion

Our exploratory RCT investigated the effects of descriptive versus simulated-experience formats on GPs’ and pain specialists’ objective risk perceptions of the benefits and harms of strong opioids (measured in terms of numerical estimates) and their prescription behaviors when treating patients with chronic noncancer pain. This investigation is the first to systematically explore a potential description–experience gap in the domain of drug safety.

Our most important finding is that descriptive and simulated-experience formats can have notably different effects on physicians’ prescription behavior. Compared to the fact box, the interactive simulation resulted in significantly lower reported prescription rates for strong WHO-III opioids—the main target of the intervention—among GPs and pain specialists at follow-up. The interactive simulation also prompted significantly greater reductions from baseline to follow-up in prescription rates of strong and weak opioids and greater increases in prescriptions of non-drug-based therapy approaches such as psychotherapy or means of opioid reduction. Furthermore, compared to the fact box, the interactive simulation triggered a stronger implementation of intended behavior [49] among GPs and pain specialists after intervention for several therapy options. At this point, we can only speculate about why physicians’ actual prescription behavior at T2 was more consistent with and sometimes even exceeded their intentions in the simulated-experience intervention compared to the fact-box intervention. Unlike static descriptions, interactive simulations empower participants to sequentially observe the occurrence and potential tapering off of a drug’s benefits as well as the emergence of serious harm over time. Perhaps insights into the otherwise difficult to detect sequential dynamics behind the benefit–harm ratio—insights that may more realistically reflect physicians’ observations of medication effects in real practice—prompt and sustain physicians’ goal of ending their patients’ long-term opioid prescription before serious harms occur. Our RCT is, to our knowledge, the first investigation of the effects of different modes of learning about risks on actual behavior and the correspondence between behavioral intention and actual behavior. It shows promising results for the field of drug safety but also leaves questions—for example, about exact mechanisms and domain specificity—unanswered. More work is needed to replicate these findings and to better understand the mechanisms and factors causing them.

The second important finding in our study was that neither format was consistently superior in improving estimates on the benefits and harms of risky drugs. We did not find systematic differences in the proportion of correct estimates on opioids’ benefits and harms between intervention groups or specialty. There were also no systematic differences in increase in the proportion of correct estimates between groups. This finding somewhat contradicts studies in other domains [34, 39] that found that the simulated-experience format was clearly more effective. One potential explanation for our finding is that health professionals are familiar with tabular presentations of risk information such as fact boxes (e.g., tables listing side effects in package leaflets), but are likely unfamiliar with interactive simulations. Given that our RCT was conducted during the COVID-19 pandemic, a particularly challenging time for health care providers, some physicians may have found it easier to attend to a familiar risk format. Further, our descriptive format—in contrast to other studies—required participants to interact with it in order to reveal the numerical estimates of opioids’ benefits and harms; similar degrees in interactivity of descriptive and simulated formats appear to help diminish different effects in fostering exact risk understanding. Last but not least, fewer than 5 percent of the 150 physicians per specialty ran the interactive simulation more than once or used filter functions, suggesting that most physicians did not harness the simulation’s full potential. Our study may therefore underestimate the potential effect of the interactive simulation on numerical risk estimates; it may also suggest that interactive simulations require time that physicians do not always have. Still, although the simulated-experience format did not produce better numerical estimates of the benefits and harms compared to the descriptive format, it did have a more desirable effect on physicians’ prescription behavior—and, by extension, likely on drug safety. The simulated-experience format may thus offer a behavioral advantage over the descriptive format, without a notable disadvantage in terms of risk knowledge.

Finally, our findings confirm the importance of medical risk literacy. Medical risk literacy can affect whether physicians amend erroneous numerical estimates: The higher their medical risk literacy, the more likely participants were to update their risk perception of strong opioids’ benefits and harms.

Our exploratory RCT has limitations. First, the results are based on convenience samples, thus potentially diminishing their generalizability. Second, we do not know why some physicians—regardless of the intervention they received—did not revise their initial estimates even though they clearly diverged from the evidence presented. Given that the information was presented in accordance with current guidelines for evidence-based health information, it is unlikely that physicians did not know how to interpret the data presented. Furthermore, there is evidence that fact boxes are effective even for laypeople with low literacy levels [29, 30]. A more likely explanation for these findings is therefore that exhaustion or time pressure during the COVID-19 pandemic limited physicians’ capacity to fully engage with the educational material.

These limitations notwithstanding, the ERONA trials with GPs and pain specialists provide initial evidence that two promising tools—one description-based and one simulated-experience-based—can be used to transparently inform physicians about the evidence surrounding a potent but high-risk drug. Our findings also suggest that one size may not fit all: A particular risk education format may be effective for some aspects and ineffective for others [53]. Physicians could therefore be offered the opportunity to select their preferred way of learning about the benefits and harms of drugs and therapies in order to help them act more in line with drug safety concerns. There is likely no format that serves all the objectives of communicating health-related information equally well. If the goal of an educational intervention is to improve physicians’ exact numerical knowledge about the benefits and harms of a risky drug, descriptive and simulated-experience formats might be equally suitable. If the goal is to boost physicians’ prescription intentions and thereby reduce or end the practice of long-term prescription of risky drugs such as strong opioids, a simulated-experience format appears to be the better choice.

## Supplementary Information

**Additional file 1:** **Supplemental materials:** CONSORT flow chart: General practitioners. CONSORT flow chart: Pain specialists. **TableS1.** Propensity to implement intended prescription behavior (T1) into actual prescription behavior (T2) per intervention. **Table S2.** Dropout analysis results for reported prescription behaviour at T0 and reported intended prescription behaviour at T1.

## Data Availability

All data generated or analyzed during the part of the study reported here are included in this published article and its supplementary material. The data set from which the results were derived can be made available to authorized individuals upon written request to the first author. Additional information will be made publicly available via the Open Science Framework under https://osf.io/swqpm/ when the ERONA project is concluded.
