# Supplementary material for: Educating physicians on strong opioids by descriptive versus simulated-experience formats: a randomized controlled trial
Source: BMC Med Educ. 2022 Oct 26;22:741. doi: 10.1186/s12909-022-03797-7 (PMC9607791; doi:10.1186/s12909-022-03797-7)
Supplement: Supplementary file 1 — Additional file 1: Supplemental materials: CONSORT flow chart: General practitioners. CONSORT flow chart: Pain specialists. TableS1. Propensity to implement intended prescription behavior (T1) into actual prescription behavior (T2) per intervention. Table S2. Dropout analysis results for reported prescription behaviour at T0 and reported intended prescription behaviour at T1. [file 12909_2022_3797_MOESM1_ESM.docx]

Supplemental Materials

Wegwarth, O., Spies, C., Ludwig, W. D., Donner-Banzhoff, N., Jonitz, G., & Hertwig, R.: **Educating Physicians on Strong Opioids by Descriptive versus Simulated-Experience Formats: A Randomized Controlled Trial.**

***Content***

1. CONSORT flow chart: General practitioners
2. CONSORT flow chart: Pain specialists
3. Table S1: **Propensity to implement intended prescription behavior (T1) into actual** prescription **behavior (T2) per intervention**
4. Table S2: Dropout analysis results for reported prescription behaviour at T0 and reported intended prescription behaviour at T1


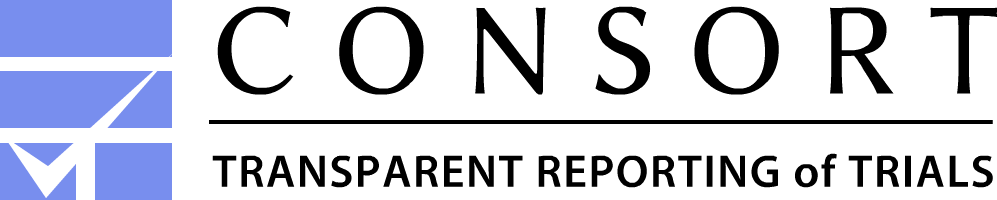


**CONSORT 2010 Flow Diagram | ERONA trial with general practitioners**

Excluded (n = 3,131)

♦  Not meeting inclusion criteria (n = 7)

♦  Did not finish the survey (n = 55)

♦  Did not respond (n = 3,069)

Lost to follow-up due to nonresponse to invitation to wave 2 (n = 46)

Analyzed for wave 1 (T1) (n = 150)

**Allocated to simulated experience intervention** (n= 150)

♦ Received allocated intervention (n = 150 )

♦ Did not receive allocated intervention

(n = 0)

Analyzed for wave 1 (T1) (n = 150)

Lost to follow-up due to nonresponse to invitation to wave 2 (n = 40)

Analyzed (n = 104)

Assessed for eligibility

(n = 3,431)

## Analysis T1

## Enrollment

Randomized (n= 300)

## Analysis T2

Analyzed (n = 110)

## Follow-up (T2)

## Allocation

**Allocated to descriptive intervention**

(n = 150)

♦ Received allocated intervention (n = 150)

♦ Did not receive allocated intervention (n = 0)


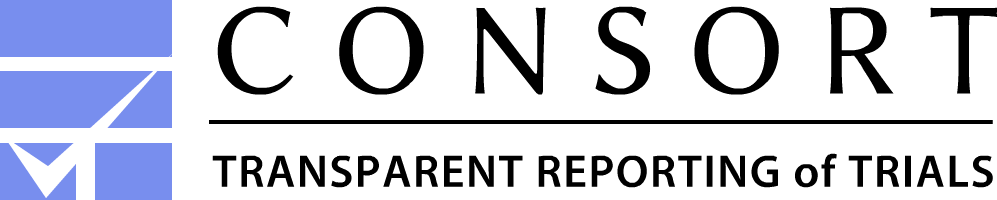


**CONSORT 2010 Flow Diagram | ERONA trial with pain specialists**

Lost to follow-up due to nonresponse to invitation to wave 2 (n = 51)

Analyzed for wave 1 (T1) (n= 150)

Assessed for eligibility

(n= 5,389)

## Enrollment

**Allocated to descriptive intervention**

(n= 150)

♦ Received allocated intervention (n= 150)

♦ Did not receive allocated intervention (n= 0 )

Randomized (n= 300)

Excluded (n= 5,089)

♦  Not meeting inclusion criteria (n= 0)

♦  Did not finish the survey (n= 72)

♦  Did not respond (n= 5,017)

## Analysis T2

Analyzed (n = 99)

## Follow-up (T2)

## Analysis T1

## Allocation

**Allocated to simulated experience intervention** (n= 150)

♦ Received allocated intervention (n= 150 )

♦ Did not receive allocated intervention (n= 0)

Analyzed for wave 1 (T1) (n= 150)

Analyzed (n= 113)

Lost to follow-up due to nonresponse to invitation to wave 2 (n = 37)

**Table S1**

***Propensity to Implement Intended Prescription Behavior (T1) Into Actual*** *Prescription* ***Behavior (T2) per Intervention***

| ***General practitioners*** | **Fact box intervention**  (*n* = 110) | | **Simulated experience intervention**  (*n* = 104) | | ***Differences between interventions*** |
| --- | --- | --- | --- | --- | --- |
| **Treatments prescribed to patients with chronic, noncancer pain** | *Actual behavior is equal or exceeds intended behavior ^§^*  *(% people)* | *Actual behavior is inferior to intended behavior^§§^*  *(% people)* | *Actual behavior is equal or exceeds intended behavior ^§^*  *(% people)* | *Actual behavior is inferior to intended behavior^§§^*  *(% people)* | ***p**** |
| Strong opioids | 93.3 | 6.7 | 98.2 | 1.8 | .013* |
| Nonsteriod anti-inflammatory drugs | 90.9 | 9.1 | 89.4 | 10.6 | .602 |
| Weak opioids | 79.1 | 20.9 | 94.2 | 5.8 | .001* |
| Multimodal therapy | 80.9 | 19.1 | 88.0 | 12.0 | .041* |
| Physiotherapy, endurance sports | 90.9 | 9.1 | 92.6 | 16.0 | .519 |
| Psychotherapy | 89.0 | 11.0 | 91.7 | 8.3 | .343 |
| Means of opioid reduction | 82.8 | 17.2 | 92.2 | 7.8 | .003* |
| ***Pain specialists*** | **Fact box intervention**  (*n* = 99) | | **Simulated experience intervention**  (*n* = 113) | |  |
| Strong opioids | 91.9 | 8.1 | 98.2 | 1.8 | .031* |
| Nonsteriod anti-inflammatory drugs | 91.9 | 8.1 | 91.2 | 8.8 | .841 |
| Weak opioids | 83.8 | 16.2 | 91.2 | 8.8 | .105 |
| Multimodal therapy | 77.8 | 22.2 | 89.4 | 10.6 | .022* |
| Physiotherapy, endurance sports | 96.0 | 4.0 | 95.6 | 4.4 | .890 |
| Psychotherapy | 93.9 | 6.1 | 92.9 | 7.1 | .766 |
| Means of opioid reduction | 86.9 | 13.1 | 95.6 | 4.4 | .023* |

*Note.* *^§^* “Exceeds intended behavior” means for the “prescription of WHO-III-opioids” and “prescription of WHO-II-opioids” a lower reported prescription rate at T2 than as intended at T1, and for the remaining 5 therapy option a higher reported prescription rate at T2 than as compared to T1. *^§§^*“Inferior to intended behavior” means for the “prescription of WHO-III-opioids” and “prescription of WHO-II-opioids” a higher reported prescription rate at T2 than as intended at T1, and for the remaining 5 therapy option a lower rate at T2 as compared to T1.

* Significance level is two-tailed and based on Chi-Square-test.

**Table S2**

*Drop-Out Analysis Results for Reported Prescription Behaviour at T0 and Reported Intended Prescription Behaviour at T1*

| ***Time of measurement*** | **T0**  **(N = 300)** | | | **T1**  **(N = 300)** | | |
| --- | --- | --- | --- | --- | --- | --- |
| ***General practitioners*** | **Non-Drop- Outs  (n = 214)**  (Mean ) | **Drop-Outs (n=86)**  Mean | ***p****  *(Cohen’s d)* | **Non-Drop- Outs (n = 214)**  (Mean ) | **Drop-Outs (n=86)**  Mean | ***p****  *(Cohen’s d)* |
| Strong opioids | 22.58 | 22.56 | .985 (.00) | 22.3 | 21.84 | .731 (.04) |
| Nonsteriod anti-inflammatory drugs | 86.15 | 85.05 | .569 (.08) | 86.38 | 85.34 | .585 (.08) |
| Weak opioids | 12.86 | 16.28 | .126 (.17) | 12.73 | 15.99 | .133 (.16) |
| Multimodal therapy | 17.39 | 16.94 | .798 (.07) | 18.07 | 17.87 | .915 (.02) |
| Physiotherapy, endurance sports | 67.61 | 65.87 | .522 (.08) | 68 | 66.74 | .611 (.06) |
| Psychotherapy | 29.51 | 26.47 | .338 (.13) | 30.27 | 27.22 | .350 (.12) |
| Means of opioid reduction | 26.78 | 23.84 | .327 (.13) | 27.82 | 24.9 | .346 (.12) |
| ***Pain specialists*** | **Non-Drop- Outs (n = 212)**  (Mean ) | **Drop-Outs (n=88)**  Mean | ***p****  *(Cohen’s d)* | **Non-Drop- Outs (n = 212)**  (Mean ) | **Drop-Outs (n=88)**  Mean | ***p****  *(Cohen’s d)* |
| Strong opioids | 26.22 | 28.3 | .149 (.18) | 25.91 | 27.78 | .197 (.16) |
| Nonsteriod anti-inflammatory drugs | 77.58 | 83.39 | .025 (.28) | 77.85 | 84.06 | .009 (.31) |
| Weak opioids | 15.54 | 13.51 | .194 (.17) | 15.22 | 12.93 | .129 (.19) |
| Multimodal therapy | 27.12 | 24.43 | .184 (.16) | 28.02 | 25.11 | .168 (.17) |
| Physiotherapy, endurance sports | 64.56 | 63.49 | .663 (.06) | 64.65 | 63.55 | .650 (.36) |
| Psychotherapy | 47.06 | 41.15 | .125 (.19) | 47.36 | 41.55 | .134 (.36) |
| Means of opioid reduction | 42.68 | 32.44 | .006 (.23) | 43.24 | 32.56 | .004 (.36) |

*Note.* * Significance level is two-tailed and based on an independent t-test.
